# ﻿A new horned toad of *Boulenophrys* (Anura, Megophryidae) was discovered in Hubei Province, China

**DOI:** 10.3897/zookeys.1253.154757

**Published:** 2025-09-26

**Authors:** Ruiqi Wang, Lu Chen, Jinliang Chen, Haochen Huang, Gangzhi Peng, Honglin Peng, Xinzhang Gao, Bangqing Chen, Naiyi Liu

**Affiliations:** 1 Hubei Broad Nature Technology Service Co., Ltd. Wuhan, 430079, China Hubei Broad Nature Technology Service Co. Wuhan China; 2 Houhe Nature Reserve Administration, Yichang, 443413, China Houhe Nature Reserve Administration Yichang China; 3 Key Laboratory of Tropical Marine Bio-resources and Ecology, South China Sea Institute of Oceanology, Chinese Academy of Sciences, Guangzhou, 510301, China South China Sea Institute of Oceanology, Chinese Academy of Sciences Guangzhou China; 4 University of Chinese Academy of Sciences, Beijing, 100049, China University of Chinese Academy of Sciences Beijing China; 5 Dalaoling Nature Reserve Administration of Yichang Three Gorges, Yichang, 443100, China Dalaoling Nature Reserve Administration of Yichang Three Gorges Yichang China; 6 Jiangsu Key Laboratory for Biodiversity and Biotechnology, College of Life Sciences, Nanjing Normal University, Nanjing, Jiangsu 210023, China Nanjing Normal University Nanjing China; 7 Department of Ecology, School of Life Sciences, Nanjing University, Nanjing, China Nanjing University Nanjing China

**Keywords:** *Boulenophrys dalaolingensis* sp. nov., horned toad, Hubei Province, new species, phylogeny, taxonomy

## Abstract

The diversity of the genus *Boulenophrys*, with around 72 species identified so far, is still thought to be highly underestimated, as it contains a large number of undescribed cryptic species. The favorable ecological environment of the Dalaoling National Nature Reserve, located in Yichang City, Hubei Province, is home to many characteristic species. This work describes a new species from central China, namely: *Boulenophrys dalaolingensis* Wang, Chen & Liu, 2025, **sp. nov.** Morphologically, the new species is characterized by the combination of nine external characters: (1) medium-sized body (SVL 49.9–56.2 mm in seven males, SVL 50.3–60.0 mm in three females; (2) vomerine teeth absent; (3) margin of tongue smooth, with weakly notch behind; (4) relative finger length III>II=I>IV; (5) tibio-tarsal articulation of adpressed limb reaching posterior corner of the eye; and (6) toes with more than 1/4 web. Molecularly, the new species forms an independent clade with strong support in the phylogenetic trees of the genus based on two partial mitochondrial sequences: 16S ribosomal RNA (16S rRNA) and cytochrome *c* oxidase subunit I (COI). Morphological and molecular phylogenetic analyses suggest that it is a new species that has not been systematically described. Our work increases the number of species in the genus *Boulenophrys* to 73.

## ﻿Introduction

Asian horned toads, belonging to the subfamily Megophryinae, encompass more than 141 species, forming a widely distributed, diverse, and complex group (Frost 2025). These amphibians inhabit tropical and subtropical montane forests across Asia, ranging from southern China and the southern and eastern Himalayas to the Indochina Peninsula, the Malay Archipelago, and as far as Wallace’s Line (Frost 2025). Due to both the morphological similarities among species and the intricate patterns of genetic divergence, the generic classification of Megophryinae has been a subject of ongoing debate (Chen et al. 2017; Mahony et al. 2017; Lyu et al. 2021; Qi et al. 2021; Lyu et al. 2023; Qian et al. 2023; Wang et al. 2024; Frost 2025). Recently, a ten-genera classification for the Asian horned toads, which also corresponds to geographic patterns, was established by Lyu et al. (2023) based on morphological and phylogenetic evidence. At present, the subfamily Megophryinae contains ten genera, namely *Atympanophrys* Tian & Hu, 1983, *Boulenophrys* Fei, Ye & Jiang, 2016, *Brachytarsophrys* Tian & Hu, 1983, *Grillitschia* Dubois, Ohler & Pyron, 2021, *Jingophrys* Lyu & Wang, 2023, *Megophrys* Kuhl & Van Hasselt, 1822, *Ophryophryne* Boulenger, 1903, *Pelobatrachus* Beddard, 1908 “1907”, *Sarawakiphrys* Lyu & Wang, 2023, and *Xenophrys* Günther, 1864. Among these, 72 species have been discovered in *Boulenophrys* (Qi et al. 2021; Lyu et al. 2023; Frost 2025), which is the most species-diverse genus in subfamily, and species of this genus continue to be discovered every year (Lin et al. 2024; Wang et al. 2024; Zeng et al. 2024).

The species diversity of *Boulenophrys* in Hubei Province, China, lacks studies. Currently, only four species of *Boulenophrys* have been recorded in the province, namely: *Boulenophrys caudoprocta* (Shen, 1994), *B. sangzhiensis* (Jiang, Ye & Fei, 2008), *B. wushanensis* (Ye & Fei, 1995) and *B. boettgeri* (Boulenger, 1899) (AmphibiaChina 2025).

During field surveys in Dalaoling National Nature Reserve, Yiling District, Hubei Province, China, we collected a number of *Boulenophrys* specimens. Molecular phylogenetic analyses and morphological comparisons support their recognition as an undescribed species.

## ﻿Material and methods

### ﻿Sampling

**Figure 1.**
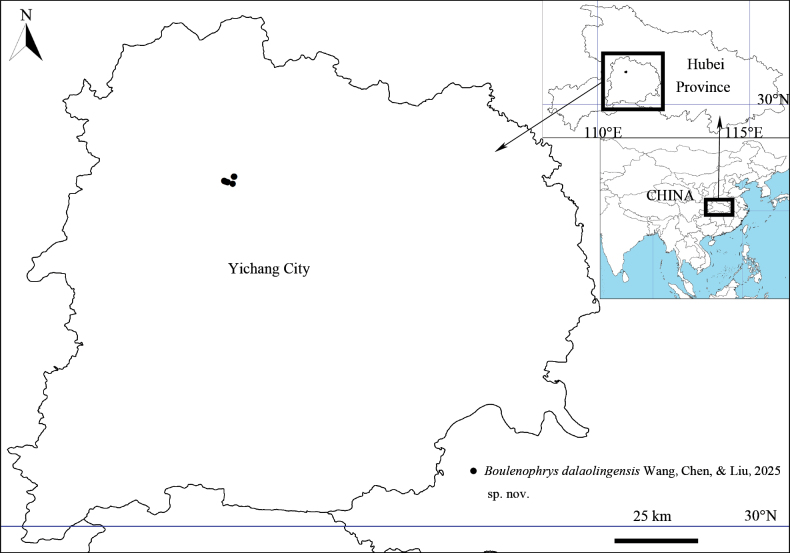
Map showing the known distribution of *Boulenophrys dalaolingensis* sp. nov. in Hubei Province, China.

A total of 10 specimens (seven adult males and three adult females) were collected from Dalaoling National Nature Reserve, Yiling District, Hubei Province, China in May 2024 (Fig. 1). All specimens were fixed in 10% buffered formalin and later transferred to 75% ethanol, and deposited in the Nanjing Normal University (NNU). Prior to fixation, tissue samples (liver or muscle) were extracted and stored in 95% ethanol, and subsequently stored at -40 °C. The procedures for DNA tissue sampling and specimen fixation follow the protocols detailed in Qi et al. (2021).

### ﻿Molecular data and phylogenetic analyses

Genomic DNA was extracted from muscle or liver tissues using a DNA extraction kit from the Tiangen Biotech (Beijing) Co., Ltd. Two partial mitochondrial sequences of 16S ribosomal RNA (16S rRNA) and cytochrome *c* oxidase subunit I (*COI*) were amplified and sequenced using the following primers: P7 (5’-CGCCTGTTTACCAAAAACAT-3’) and P8 (5’-CCGGTCTGAACTCAGATCACGT-3’) for 16S rRNA (Simon et al. 1994), Chmf4 (5’-TYTCWACWAAYCAYAAAGAYATCGG-3’) and Chmr4 (5’-ACYTCRGGRTGRCCRAARAATCA-3’) for *COI* (Che et al. 2012). PCR was performed using the primers and conditions specified by Liu et al. (2018). The PCR products were purified using spin columns and sequenced in both directions on an ABI 3730 automated genetic analyzer by Tianyi Huiyuan Biotech (Wuhan) Co., Ltd. All the newly generated nucleotide sequences were initially assembled and manually edited using DNASTAR Lasergene 7.1. Newly sequenced samples were deposited in GenBank (Table 1).

**Table 1.** Scientific name, location, voucher information and GenBank accession numbers of the species of genus *Boulenophrys* selected for this study.

ID	Genus/Species	Locality	Voucher ID	16S rRNA	*COI*	Reference
1	*Boulenophrys dalaolingensis*	Dalaoling Nature Reserve, Hubei, China	NNUYC240506	PV816345	PV814947	this study
2	*Boulenophrys dalaolingensis*	Dalaoling Nature Reserve, Hubei, China	NNUYC240507	PV816346	PV814945	this study
3	*Boulenophrys dalaolingensis*	Dalaoling Nature Reserve, Hubei, China	NNUYC240510	PV816347	PV814946	this study
1	*Boulenophrys anlongensis*	Anlong, Guizhou, China	CIB AL20190531018	MT823184	MT823261	Li et al. 2020
2	*Boulenophrys anlongensis*	Anlong, Guizhou, China	CIB AL21090531017	MT823185	MT823262	Li et al. 2020
3	*Boulenophrys binchuanensis*	Mt. Jizu, Yunnan, China	SYS a007843	OQ180976	OQ180864	Lyu et al. 2023
4	*Boulenophrys binchuanensis*	Mt. Jizu, Yunnan, China	KIZ 019442	KX811850	KX812113	Chen et al. 2017
5	*Boulenophrys binlingensis*	Mt. Wawu, Sichuan, China	KIZ 025807	KX811852	KX812115	Chen et al. 2017
6	*Boulenophrys binlingensis*	Mt. Wawu, Sichuan, China	SYS a005313	MH406892	MH406354	Liu et al. 2018
7	*Boulenophrys daweimontis*	Xichou, Yunnan, China	KIZ 016066	KX811866	KX812124	Chen et al. 2017
8	*Boulenophrys daweimontis*	Mt. Dawei, Yunnan, China	KIZ 048938	KX811870	KX812126	Chen et al. 2017
9	*Boulenophrys fanjingmontis*	Mt. Fanjing, Guizhou, China	SYS a004350	MH406808	MH406270	Liu et al. 2018
10	*Boulenophrys jingdongensis*	Mt. Cenwanglaoshan, Guangxi, China	SYS a005160	MH406883	MH406345	Liu et al. 2018
11	*Boulenophrys jingdongensis*	Mt. Wuliang, Yunnan, China	SYS a003928	MH406773	MH406232	Liu et al. 2018
12	*Boulenophrys lushuiensis*	Lushui County, Yunnan, China	CIB YN201909288	MW001225	MW000912	Shi et al. 2021
13	*Boulenophrys omeimontis*	Chishui, Guizhou, China	SYS a004939	MH406866	MH406328	Liu et al. 2018
14	*Boulenophrys omeimontis*	Mt. Emei, Sichuan, China	SYS a005301	MH406887	MH406349	Liu et al. 2018
15	*Boulenophrys palpebralespinosa*	Pu Hu, Thanh Hoa, Vietnam	KIZ 011650	KX811889	KX812138	Chen et al. 2017
16	*Boulenophrys qianbeiensis*	Yibin, Sichuan, China	WG202107005	ON552245	ON565473	Song et al. 2023
17	*Boulenophrys qianbeiensis*	Tongzi, Guizhou, China	CIB TZ20190608017	MT651554	MT654521	Su et al. 2020
18	*Boulenophrys rubrimera*	Jinping, Yunnan, China	KIZ 020425	KX811871	KX812123	Chen et al. 2017
19	*Boulenophrys rubrimera*	Sapa, Lao Cai, Vietnam	AMS R177676	MF536419	MW086542	Tapley et al. 2020
20	*Boulenophrys sangzhiensis*	Shimen, Hunan, China	SYS a008386	OQ180982	OQ180870	Lyu et al. 2023
21	*Boulenophrys sangzhiensis*	Zhangjiajie, Hunan, China	SYS a004306	MH406797	MH406259	Liu et al. 2018
22	*Boulenophrys spinata*	Mt. Leigong, Guizhou, China	KIZ 016100	KX811864	KX812119	Chen et al. 2017
23	*Boulenophrys spinata*	Mt. Leigong, Guizhou, China	SYS a002226	MH406675	MH406115	Liu et al. 2018
24	*Boulenophrys wuliangshanensis*	Mt. Ailao, Yunnan, China	SYS a002983	MH406730	MH406182	Liu et al. 2018
25	*Boulenophrys wuliangshanensis*	Mt. Wuliang, Yunnan, China	SYS a003924	MH406771	MH406230	Liu et al. 2018
26	*Boulenophrys wushanensis*	Mt. Guangwu, Sichuan, China	KIZ 045469	KX811838	KX812094	Chen et al. 2017
27	*Boulenophrys wushanensis*	Mt. Wu, Hubei, China	SYS a003008	MH406732	MH406184	Liu et al. 2018
28	*Boulenophrys tuberogranulata*	Badagongshan Nature Reserve, Hunan, China	SYS a004310	MH406801	MH406263	Liu et al. 2018
29	*Xenophrys mangshanensis*	Huaiji, Guangdong, China	SYS a002177	MH406666	MH406106	Liu et al. 2018
30	*Xenophrys glandulosa*	Mt. Gaoligong, Yunnan, China	SYS a003757	MH406754	MH406213	Liu et al. 2018

For phylogenetic analysis, 25 homologous sequences of *Boulenophrys omeimontis* species group, three sequences of other *Boulenophrys* species group and two outgroup taxa (*Xenophrys glandulosa* (Fei, Ye & Huang, 1990) and *X. mangshanensis* (Fei & Ye, 1990)) were downloaded from GenBank (Table 1). Our final dataset included 18 described species of *Boulenophrys* (14 described species of *Boulenophrys omeimontis* species group) and *Xenophrys*. DNA sequences were aligned in MEGA 7 (Kumar et al. 2016) by the Clustal W algorithm with default parameters (Thompson et al. 1997). The GTR+G+I model was selected as the best model for each partition by MEGA 7 (Kumar et al. 2016). Phylogenetic trees were constructed using maximum likelihood (ML) and Bayesian inference (BI) implemented in MEGA 7 (Kumar et al. 2016) and MrBayes 3.2.5 (Ronquist et al. 2012). Four independent runs were conducted during the BI analyses with 10,000,000 generations each, and they were sampled every 1000 generations, with the first 25% of samples discarded as burn-in, resulting in a potential scale reduction factor (PSRF) of <0.005. Mean genetic distances between and within species were calculated using uncorrected pairwise distances (*p*-distance) by *COI* implemented in MEGA 7 (Kumar et al. 2016).

### ﻿Morphology

Morphological measurements from preserved specimens were conducted as described by Lyu et al. (2021) using a digital caliper (to the nearest 0.1 mm). The measurements were as follows : snout-vent length (**SVL**): from tip of snout to vent ; head length (**HDL**): from tip of snout to rear of jaws ; head width (**HDW**): head width at commissure of jaws ; snout length (**SNT**): from tip of snout to anterior corner of eye ; internasal distance (**IND**): distance between nares ; interorbital distance (**IOD**): minimum distance between middle upper eyelids ; eye diameter (**ED**): diameter of exposed portion of eyeball ; tympanum diameter (**TD**): horizontal diameter of tympanum ; tympanum-eye distance (**TED**): distance from anterior edge of tympanum to posterior margin of eye ; hand length (**HND**): distance from distal end of radioulna to tip of phalanx of finger III ; radioulna length (**RAD**) ; tibia length (**TIB**): distance from knee to heel, flexed at 90° ; foot length (**FTL**): distance from distal end of tibia to tip of distal phalanx of toe IV. Morphological descriptions follow the definitions by Lyu et al. (2021). Sex was determined by the presence of nuptial pads/spines in males and/or gonadal inspection.

## ﻿Results

**Figure 2.**
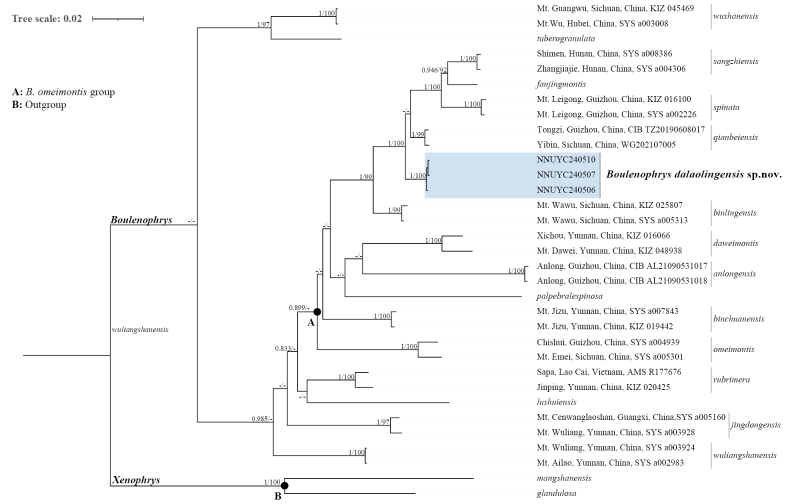
Phylogram of *B. omeimontis* species group based on the 16S and *COI* genes. “-” denotes Bayesian posterior probabilities (BPP) < 0.8 and bootstrap support (BS) < 80. New samples for the present study are indicated in bold font.

The ML and BI analyses recovered similar tree topologies with strong nodal support, differing mainly at terminal nodes identified as weakly supported or collapsed. The *Boulenophrys omeimontis* group (Fig. 2, Node A) was recovered as monophyletic with weak support from both analyses (BPP=0.899; Fig. 2). The newly collected samples from Dalaoling Nature Reserve, Hubei, China, clustered into a monophyletic clade with strong nodal support (BPP=1; BS=100). This clade clustered with *B. sangzhiensis*, *B. spinata*, *B. qianbeiensis*, and *B. binlingensis* with strong support (BPP=1; BS=100).

The mean uncorrected *p*-distances based on *COI* among all in-group species used in this study are given in Appendix 1. The genetic distance between the new species and the closest related species, *B. qianbeiensis*, was about 2.50%–2.51%. It is comparable to the divergences among the nearest neighbor genetic distances of this group, which ranged from 3.21% (*B. sangzhiensis* and *B. fanjingmontis*) to 4.64% (*B. qianbeiensis* and *B. spinata*) for *COI*.

Moreover, morphologically these specimens can be distinguished from the *B. omeimontis* group by a unique combination of morphological features (see Taxonomic account below). Thus, we describe these specimens as a new species of the *B. omeimontis* group.

### ﻿Taxonomic account

#### ﻿ Boulenophrys dalaolingensis

Taxon classificationAnimaliaAnuraMegophryidae

Wang, Chen & Liu, 2025 sp. nov.

A0D3CEBD-2334-5456-B8AB-18FB8080D35E

https://zoobank.org/42A00F8E-1FF1-42D0-A091-EB7FF9060F07

##### Type material.

***Holotype*.** NNUYC240510, adult male, collected on 12 May 2024 by Ruiqi Wang, Lu Chen, Gangzhi Peng, Honglin Peng, Xinzhang Gao from the Dalaoling Nature Reserve, Yiling District, Yichang City, Hubei Province, China (31.0630°N, 110.9249°E; elevation 1629 m a.s.l.).

***Paratypes*.** Six adult males (NNUYC240501, NNUYC240503, NNUYC240505, NNUYC240507, NNUYC240508, and NNUYC240509) and three adult females (NNUYC240502, NNUYC240504, and NNUYC240506) collected at the same locality and with the same collection information as the holotype.

##### Etymology.

The specific epithet “*dalaolingensis*” is a Latinized adjective derived from the name of Dalaoling Nature Reserve, Hubei Province, China, which is the type locality of this species. We propose the English common name “Mt. Dalaoling Horned Toad” and the Chinese common name “Dà Lǎo Lǐng Jiǎo Chán (大老岭角蟾)”.

##### Diagnosis

**Figure 3.**
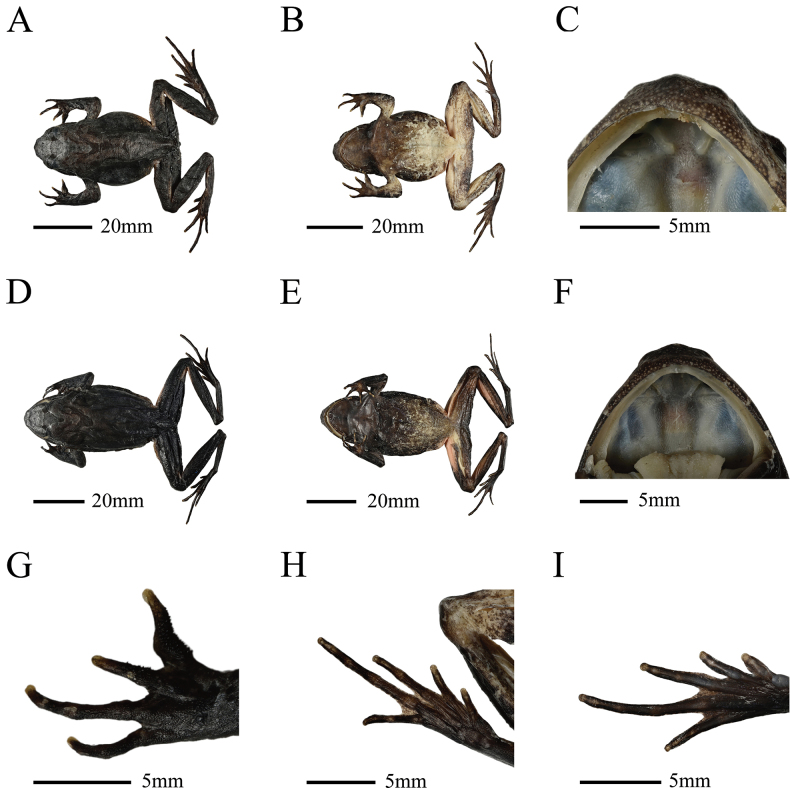
*Boulenophrys dalaolingensis* sp. nov. in preservative. A. Holotype (NNUYC240510), dorsolateral view, male; B. Holotype (NNUYC240510), ventral view; C. Holotype (NNUYC240510), oral view; D. Paratype (NNUYC240506), dorsolateral view, female; E. Paratype (NNUYC240506), ventral view; F. Paratype (NNUYC240506), oral view; G. Holotype (NNUYC240510), forelimb; H. Holotype (NNUYC240510), hindlimb; I. Paratype (NNUYC240506), hindlimb.

**Figure 4.**
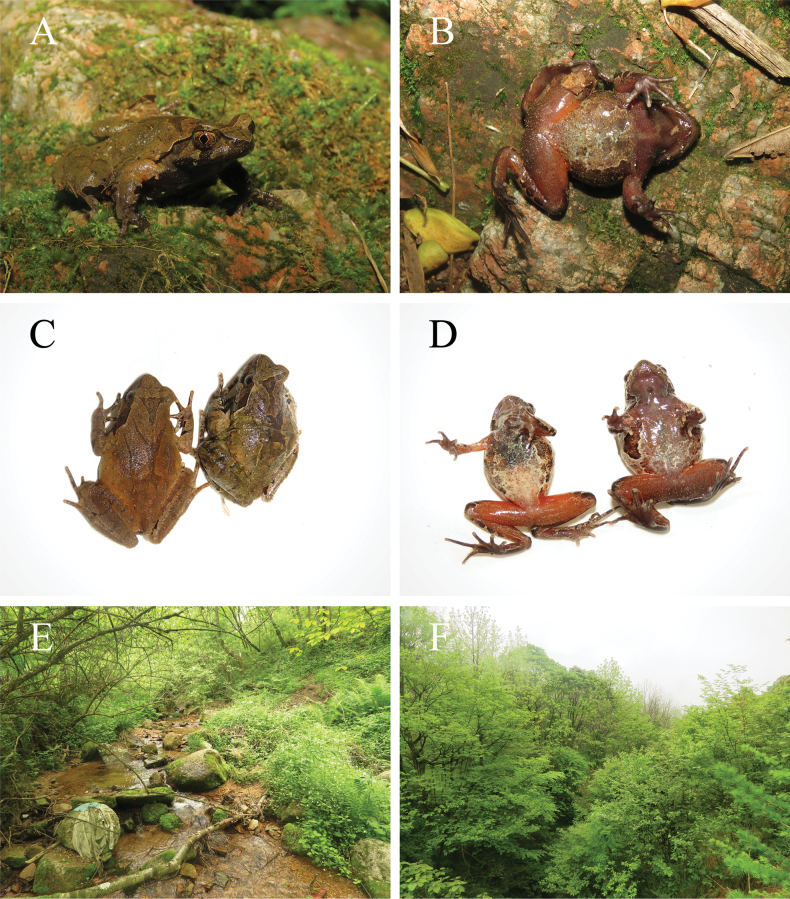
*Boulenophrys dalaolingensis* sp. nov. in life. A, B. Holotype (NNUYC240510), male; C. Paratype, dorsolateral view (right: NNUYC240503, male; left: NNUYC240504, female); D. Paratype, ventral view (right: NNUYC240504, female; left: NNUYC240503, male); E, F. Habitat.

**(Table 2, Figs 3, 4).** The new species is recognized as a member of the *B. omeimontis* group based on molecular phylogenetic analyses and can be distinguished from its groups by a combination of the following characters: (1) medium-sized body, SVL 49.9–56.2 mm in seven males, SVL 50.3–60.0 mm in three females; (2) head width larger than head length, or nearly equal; (3) vomerine ridge without obvious V-shaped and vomerine teeth absent; (4) supratympanic fold prominent; (5) small nodules on the back, forming a weak V-shaped ridge; two discontinuous dorsolateral parallel ridges on each side of the V-shaped ridge; (6) margin of tongue smooth, with weakly notch behind; (7) maxillary teeth developed; (8) relative finger length III>II=I>IV; (9) tibio-tarsal articulation of adpressed limb reaching the posterior corner of eye; (10) iris reddish brown, black reticulations throughout, pupils vertical; (11) toes with more than 1/4 web; (12) lateral fringes of toes wide in males and not significant in females; and (13) males with gray nuptial pads at the base of the first and second fingers, covered thick and dense black nuptial spines.

**Table 2.** Morphological measurements of the type series of *Boulenophrys dalaolingensis* sp. nov. The asterisk (*) indicates the holotype.

Number	NNUYC 240501	NNUYC 240502	NNUYC 240503	NNUYC 240504	NNUYC 240505	NNUYC 240506	NNUYC 240507	NNUYC 240508	NNUYC 240509	NNUYC 240510*
Sex	Male	Female	Male	Female	Male	Female	Male	Male	Male	Male
SVL	50.8	55.2	50.0	50.3	51.0	60.0	56.2	52.9	50.3	49.9
HDL	16.9	16.7	16.2	14.8	15.3	18.3	16.7	15.9	15.0	15.0
HDW	18.3	17.3	16.6	16.3	17.9	18.9	18.5	17.5	16.8	17.5
SNT	4.4	4.7	4.5	3.9	4.8	4.3	4.5	5.0	5.2	3.6
IND	6.1	5.5	5.7	4.7	5.2	7.0	5.6	5.4	5.5	6.3
IOD	4.8	4.5	3.9	4.0	4.5	6.1	4.6	4.6	4.4	5.1
ED	5.2	5.3	5.2	4.4	5.7	5.9	4.5	4.7	5.2	5.0
TD	2.9	2.8	2.7	3.0	3.7	3.5	3.2	3.4	3.4	2.7
TED	2.9	2.5	2.8	2.5	2.9	2.8	2.7	2.9	2.3	2.5
HND	21.3	22.5	21.0	19.8	21.7	24.2	23.7	21.9	22.3	22.2
RAD	10.7	11.1	11.9	10.5	11.2	11.2	11.0	10.7	11.1	11.3
TIB	25.4	25.5	24.1	24.6	25.1	28.4	28.7	26.0	27.2	25.9
FTL	21.3	22.0	21.5	21.6	24.5	26.2	26.5	23.8	23.0	22.8
ED/TD	1.79	1.89	1.93	1.47	1.54	1.69	1.41	1.38	1.53	1.85
TIB/SVL	0.50	0.46	0.48	0.49	0.49	0.47	0.51	0.49	0.54	0.52
FTL/SVL	0.42	0.40	0.43	0.43	0.48	0.44	0.47	0.45	0.46	0.46

##### Description of holotype

**(measurements in Table 2).** NNUYC240510, mature male, medium-sized body (SVL 49.9 mm); head width larger than head length (HWD/HDL 1.2); snout blunt and pointed, extending significantly beyond mandibular margin on ventral view; loreal region vertical and concave; top of head flat, viewed from the back; canthus rostralis angular; eyes large (ED/HDL 0.3); eye less than twice as long as maximum tympanum diameter (ED/TD 2.0) and shorter than snout length (SNT 3.6 mm, ED/SNT 1.4); tympanum distinct, circular in shape, relatively small (TD/HDL 0.2), with upper border concealed by supratympanic ridge; pupil vertical, near diamond-shaped; eye-tympanum distance (TYE 2.7 mm) longer than tympanum diameter (TD 2.5 mm); nostril rounded, laterally positioned, internarial distance greater than interorbital distance (IND/IOD 1.2); choanae oval; vomerine teeth absent; margin of tongue rounded, slightly notch posteriorly; pineal ocellus absent; maxillary teeth present; supratympanic fold distinct, from posterior corner of eye to above insertion of arm; male with an internal single subgular vocal sac.

Forelimbs thick and robust, HND 22.2 mm, 44% of SVL; relative finger length III>II=I>IV; fingertips round; hand lacking webbing, lateral dermal fringes absent; subarticular tubercles present at the base of each finger; two metacarpal tubercles, prominent, oval, the inner metacarpal tubercle larger than the outer one.

Hindlimbs slender, TIB (25.9 mm) greater than FTL (22.8 mm); heels slightly overlapped when flexed hindlimbs held at right angles to body axis; tibio-tarsal articulation reaching to posterior conner of eye when hindlimb stretched along body; the tibia was longer than the thigh; relative toe length I<II<V<III<IV; tip of toe round, slightly enlarged; subarticular tubercles present at the base of each toe; supernumerary tubercles absent; toes with more than 1/4 web; lateral dermal fringes on toes distinct, wide; inner metatarsal tubercle oval; no outer metatarsal tubercle.

Dorsal skin rough, numerous granules with black spines; several large warts scattered in the flank; small nodules on the back, forming a weak V-shaped ridge; two discontinuous V-shaped ridge present, its two sides extending posteriorly from above tympanum, terminating beyond level of axilla; an inverted triangle between the eyes; several small tubercles on the flank, thigh, and back of the tibia; supratympanic fold prominent; ventral surface smooth, covered with many white granules; pectoral glands indistinct; femoral glands in the posterior part of the femur and small white dermal asperities on the lateral part of the femur; the posterior end of the body clearly prominent, and an arc-shaped bulge formed by warts above the anus.

##### Coloration of holotype.

In life, dorsal surface of body olive green, inverted triangular brown spots between eyes; dorsal V-shaped ridge with brown spots; femoral and tibial dorsal transverse bands; several dark brown and white longitudinal lines on upper and lower lip; surface of abdomen grayish white with dark brown marbling; surface of ventral hind limbs bright red with many white granules; the ventral view of hand, foot and toe tip purplish-gray; femoral glands white. In preservative, dorsal surface of body faded to dark brown; the posterior part of the ventral body, the inner thighs and the upper tibia faded to milky white.

##### Secondary sex characteristics and variation.

The male with an internal single subgular vocal sac and a gray nuptial pad with large and dense nuptial spines on it at the base of the first and second fingers. In three female specimens (NNUYC240502, NNUYC240504, NNUYC240506), dorsal surface of body showed a remarkable reddish-brown color, with inverted triangular brick-red spots between the eyes and brick-red spots on the back; the crotch, inner thighs and upper tibia are bright rose red, which fades to light pink after immersion. All female specimens had narrower lateral dermal fringes of the toe compared with males.

##### Comparisons.

The new species was compared with the other species of the *B. omeimontis* species group. Based on molecular phylogenetics, *B. dalaolingensis* sp. nov. is most closely related to *B. spinata*, *B. qianbeiensis* and *B. sangzhiensis* (Rao and Yang 1997; Fei et al. 2009; Tapley et al. 2017; Li et al. 2020; Su et al. 2020; Lyu et al. 2023;).

*Boulenophrys dalaolingensis* sp. nov. is significantly different from *B. omeimontis* by the smaller body size, SVL 49.9–56.2 mm in seven males, SVL 50.3–60.0 mm in three females (vs. SVL 52.1–62.0 mm in ten males and SVL 65.7–71.2 mm in seven females), no vomerine teeth (vs. vomerine teeth present) and males with 1/3 webbing and wide lateral fringes (vs. narrow lateral fringes); from *B. sangzhiensis* by the liquid-preserved specimens having smooth vomerine ridges, vomerine ridges not obvious (vs. vomerine ridges weak, vomerine teeth absent), toes of male with 1/3 web (vs. toes with rudimentary webbing), and lateral fringes of toes wide (vs. lateral fringes absent); from *B. qianbeiensis* by vomerine teeth absent (vs. present); from *B. spinata* by the tibio-tarsal articulation joint reaching between the tympanum and the eye or not extending beyond the anterior angle of the eye (vs. reaching anterior corner of eye), relative finger length IV<I=II<III (vs. I<II<IV<III); from *B. fanjingmontis* by obvious subarticular nodules at the base of each finger (vs. distinct subarticular tubercle at base of finger I, absent on other fingers), and large and dense nuptial spines at the base of the first and second fingers (vs. sparse tiny black nuptial spines on dorsal bases of fingers I and II in breeding adult males).

*Boulenophrys dalaolingensis* sp. nov. is also significantly different from *B. anlongensis* by the larger body size, SVL 49.9–56.2 mm in seven males, SVL 50.3–60.0 mm in three females (vs. SVL 40.0–45.5 mm in four males, SVL 48.9–51.2 mm in three females), and margin of tongue smooth, slightly notched posteriorly (vs. tongue not notched posteriorly); from *B. binchuanensis* by the larger body size, SVL 49.9–56.2 mm in seven males, SVL 50.3–60.0 mm in three females (vs. SVL 34.4–36.3 mm in two males, SVL 39.4–46.2 mm in three females), margin of tongue smooth, with slightly notch behind (vs. tongue not notched or weakly notched posteriorly), lateral dermal fringes on toes distinct, wide (vs. narrow), and vomerine ridge without obvious V-shape and vomerine teeth absent (vs. absent), and margin of tongue smooth, with slight notch at the posterior tip (vs. vomerine ridge absent); from *B. binlingensis* by the larger body size, SVL 49.9–56.2 mm in seven males and SVL 50.3–60.0 mm in three females (vs. SVL 49.9–56.2mm in seven males and SVL 45.1–51.0 in three females), obvious subarticular tubercles at the base of each finger (vs. distinct subarticular tubercles at base of finger I, absent on other fingers), relative finger length IV<I=II<III (vs. II=IV<I<III), and tibio-tarsal articulation reaching to the posterior conner of the eye (vs. reaching region between center of eye and nostril); from *B. jingdongensis* by males with dense black nuptial spines on the dorsal bases of fingers I and II (vs. dense nuptial spines on dorsal bases of fingers I and II), vomerine teeth absent (vs. vomerine ridges and vomerine teeth present), and relative finger length IV<I=II<III (vs. II<I=IV<III); from *B. lushuiensis* by the larger body size, SVL 49.9–56.2 mm in seven males, SVL 50.3–60.0 mm in three females (vs. SVL 32.3–38.0 mm in 13 males and SVL 37.8–47.4 mm in 20 females), obvious subarticular tubercles at the base of each finger (vs. indistinct subarticular tubercle at bases of fingers I and II, absent on other fingers), and large dense black nuptial spines on the dorsal bases of fingers I and II (vs. tiny dense nuptial spines on dorsal bases of fingers I and II); from *B. palpebralespinosa* by the larger body size, SVL 49.9–56.2 mm in seven males (vs. SVL 36.2–38.0 mm in two males), smoother dorsal skin (vs. dorsal skin rough with large tubercles and distinct ridges), outer margin of upper eyelid with very small horn-like, prominent tubercles (vs. outer margin of upper eyelid with several large and prominent, horn-like tubercles), and large and dense black nuptial spines on dorsal bases of fingers I and II (vs. tiny nuptial spines on dorsal bases of fingers I and II); from *B. rubrimera* by the larger body size, SVL 49.9–56.2 mm in seven males (vs. SVL 26.7–30.5 mm in seven males), vomerine teeth absent (vs. present), two metacarpal tubercles, prominent (vs. metacarpal tubercles absent), relative finger length IV<I=II<III (vs. relative finger lengths I<II<IV<III), toes with more than 1/4 web, lateral dermal fringes on toes distinct, wide (vs. toes without webbing but with narrow lateral fringes); and from *B. wuliangshanensis* by the larger body size, SVL 49.9–56.2 mm in seven males, SVL 50.3–60.0 mm in three females (vs. SVL 25.9–28.5 mm in four males and SVL 32.1–38.0 mm in five females), vomerine ridge without obvious V-shaped and vomerine teeth absent (vs. vomerine ridges and vomerine teeth absent), toes with more than 1/4 web (vs. toes without webbing), and lateral dermal fringes on toes distinct, wide (vs. lateral fringes absent).

##### Distribution and ecology.

Currently, *Boulenophrys dalaolingensis* sp. nov. is known only from Dalaoling Nature Reserve, Hubei, China. The habitat is in a stream under a mixed montane subtropical coniferous and broadleaved forest at elevations between 1380–1650 m (Fig. 4). The stream is very cold, more than a meter wide, with a gritty bottom. This species is sympatrically distributed with *Liua shihi*, *B. wushanensis*, *Nanorana quadranus*, and *Bufo gargarizans*. During the field survey, males were heard in April and May with continuous calls around rocks and plants in streams, which could be heard both day and night. At night, females were observed crawling and swimming near the streams where males were active, but no mating behavior was observed. Tadpoles and eggs of this species were not found.

## ﻿Discussion

The discovery of the new species reveals a previously underestimated diversity of amphibian species in Hubei Province. Prior to this publication, only four species of *Boulenophrys* were found in Hubei Province (AmphibiaChina 2025; Frost 2025; Li et al. 2025), but there are still new species that have not been described.

The lack of follow-up surveys can pose issues regarding the listing of endangered species. *Boulenophrys dalaolingensis* is currently known only from the Dalaoling Nature Reserve, Yichang City, southwestern Hubei. The development of tourism facilities and the destruction of stream habitats have gradually affected and threatened the habitats of the new species. Therefore, there is an urgent need to obtain more data (i.e., distribution, population size, potential and existing risk factors, etc.) from long-term and extensive surveys to assess their endangered status.

Many *Boulenophrys* species have limited dispersal capacity and narrow ecological niches, leading to restricted ranges (Wang et al. 2019; Lyu et al. 2023). They are highly morphologically conserved (Qi et al. 2021; Lyu et al. 2023; Lin et al. 2024; Zeng et al. 2024) and the lack of follow-up investigations has contributed to inadequate protection (Zeng et al. 2024).

At the same time, we recorded typical sexual dimorphism in this species. The morphological differences between the two sexes of this species include not only body size and secondary sexual characteristics such as vocal sac and nuptial spine, but also body color, and the lateral fringes of the toes of females are different from those of males. Given that many species of *Boulenophrys* lack descriptions of one sex, it is necessary to conduct more extensive research on the species to clarify whether this phenomenon is common in the genus.

## Supplementary Material

XML Treatment for Boulenophrys dalaolingensis

## ﻿Additional information

### Use of AI

No use of AI was reported.

### Author ORCIDs

Ruiqi Wang https://orcid.org/0009-0001-2217-6459

Haochen Huang https://orcid.org/0009-0000-6603-3874

### Data availability

All of the data that support the findings of this study are available in the main text.

## ﻿Appendix 1

**Table A1.** Average uncorrected *p*-distances (percentage) among *Boulenophrys* species calculated from *COI* gene sequences.

ID	Species	1	2	3	4	5	6	7	8	9	10	11	12	13	14	15	16	17	18	19	20	21	22
1	*B. dalaolingensis*_NNUYC240510																						
2	*B. dalaolingensis_*NNUYC240506	0.000%																					
3	*B. dalaolingensis_*NNUYC240507	0.000%	0.000%																				
4	*B. anlongensis*_MT823261	11.765%	11.765%	11.765%																			
5	*B. binchuanensis*_KX812113	7.130%	7.130%	7.130%	11.052%																		
6	*B. binglingensis_*MH406354	4.991%	4.991%	4.991%	10.160%	6.774%																	
7	*B. daweimontis_*KX812124	9.804%	9.804%	9.804%	11.230%	9.447%	8.734%																
8	*B. fanjingmontis_*MH406270	5.526%	5.526%	5.526%	13.725%	10.160%	7.487%	12.478%	12.121%														
9	*B. jingdongensis_*MH406232	10.339%	10.339%	10.339%	11.765%	9.269%	9.269%	9.982%	10.339%	13.369%													
10	*B. lushuiensis_*MW000912	10.695%	10.695%	10.695%	12.656%	10.517%	10.873%	11.765%	11.230%	12.478%	11.052%												
11	*B. omeimontis_*MH406328	9.091%	9.091%	9.091%	13.369%	8.378%	8.200%	11.052%	10.517%	10.873%	11.586%	11.586%											
12	*B. palpebralespinosa_*KX812138	9.982%	9.982%	9.982%	13.012%	9.804%	9.447%	11.765%	11.765%	11.052%	12.299%	11.943%	11.943%										
13	*B. qianbeiensis_*ON565473	2.496%	2.496%	2.496%	11.765%	6.774%	4.635%	9.447%	9.447%	4.991%	10.873%	11.230%	9.269%	10.160%									
14	*B. qianbeiensis_*MT654521	2.509%	2.509%	2.509%	12.007%	6.631%	4.659%	9.677%	9.677%	5.018%	10.753%	10.932%	9.140%	10.394%	0.358%								
15	*B. rubrimera_*MW086542	7.665%	7.665%	7.665%	10.695%	6.952%	6.952%	9.626%	8.734%	9.626%	8.913%	9.269%	10.339%	9.804%	7.843%	7.706%							
16	*B. sangzhiensis*_OQ180870	4.456%	4.456%	4.456%	12.299%	8.378%	6.774%	10.873%	10.695%	3.209%	11.586%	10.873%	10.160%	10.873%	4.278%	4.301%	9.091%						
17	*B. spinata*_MH406115	4.991%	4.991%	4.991%	13.725%	9.091%	7.665%	11.765%	11.586%	4.278%	12.121%	12.834%	11.052%	11.052%	4.635%	4.659%	9.626%	3.565%					
18	*B. wuliangshanensis_*MH406230	9.091%	9.091%	9.091%	12.121%	8.021%	9.447%	9.982%	9.804%	11.586%	9.626%	9.982%	10.873%	11.586%	8.734%	8.423%	7.308%	9.804%	10.517%				
19	*B. wushanensis_*MH406184	12.299%	12.299%	12.299%	14.260%	11.408%	11.052%	12.121%	11.943%	14.439%	11.586%	12.299%	13.012%	13.191%	11.765%	11.828%	10.873%	12.834%	13.725%	11.230%			
20	*B. tuberogranulata_*MH406263	13.012%	13.012%	13.012%	15.330%	13.369%	11.408%	14.260%	14.795%	14.617%	13.012%	13.369%	13.012%	15.152%	12.478%	12.186%	11.765%	13.369%	14.260%	12.834%	6.952%		
21	*Xenophrys glandulosa*_MH406214	17.112%	17.112%	17.112%	18.182%	15.865%	15.865%	15.865%	15.865%	18.538%	16.043%	16.578%	17.291%	17.291%	16.578%	16.487%	15.686%	17.647%	17.291%	15.330%	18.004%	15.865%	
22	*X. mangshanensis*_MH406106	16.756%	16.756%	16.756%	18.895%	16.399%	16.934%	16.578%	16.578%	18.360%	14.973%	17.112%	15.865%	18.360%	16.578%	16.667%	17.112%	17.825%	18.182%	16.756%	17.112%	16.043%	10.873%
